# Prioritizing Chinese medicine clinical research questions in cancer palliative care from patient and caregiver perspectives

**DOI:** 10.1111/hex.13289

**Published:** 2021-06-09

**Authors:** Charlene H. L. Wong, Wendy Wong, Wai Ling Lin, David K. Y. Au, Justin C. Y. Wu, Ting Hung Leung, Irene X. Y. Wu, Vincent C. H. Chung

**Affiliations:** ^1^ Department of Medicine and Therapeutics The Chinese University of Hong Kong Shatin Hong Kong; ^2^ Jockey Club School of Public Health and Primary Care The Chinese University of Hong Kong Shatin Hong Kong; ^3^ National Institution of TCM Constitution and Preventive Medicine Beijing University of Chinese Medicine Beijing China; ^4^ Yat Hei Hong Kong Company Limited Central Hong Kong; ^5^ Hong Kong Institute of Integrative Medicine The Chinese University of Hong Kong Shatin Hong Kong; ^6^ School of Chinese Medicine The Chinese University of Hong Kong Shatin Hong Kong; ^7^ Department of Epidemiology and Health Statistics Xiangya School of Public Health Central South University Changsha China

**Keywords:** palliative care, patient participation, research priorities, stakeholder participation, traditional chinese medicine

## Abstract

**Background:**

Chinese medicine (CM) modalities, including acupuncture and Chinese herbal medicine (CHM), are popular palliative interventions among patients with cancer, but further clinical research is required to assess their effectiveness and safety.

**Objective:**

To prioritize top ten important CM clinical research questions from patients with cancer, cancer survivors and caregivers’ perspectives via a face‐to‐face prioritization workshop in Hong Kong.

**Methods:**

A list of 25 CM clinical research questions for cancer palliative care, which were identified from existing systematic reviews (SRs) and overview of SRs, was presented to 17 participants (patients with cancer [n = 5], cancer survivors [n = 6] and caregivers [n = 6]). The participants were then invited to establish consensus on prioritizing top ten research questions.

**Results:**

Among the top ten priorities, five (50%) focused on acupuncture and related therapies, while five (50%) were on CHM. The three most important research priorities were (i) manual acupuncture plus opioids for relieving pain; (ii) CHM for improving quality of life among patients receiving chemotherapy; and (iii) concurrent use of CHM plus loperamide for reducing stomatitis.

**Conclusion:**

The top ten participant‐endorsed CM clinical research priorities for cancer palliative care can guide local researchers on future direction. They can also inform local research funders on patient‐centred allocation of limited funding. Under limited research funding, the most important co‐prioritized research question from professional and patient perspectives may be addressed first.

**Patient or Public Contribution:**

Patients with cancer, cancer survivors and caregivers participated in conduct of the study to prioritize CM clinical research questions.

## 1 INTRODUCTION

Cancer is a major contributor to global burden of non‐communicable diseases. The World Health Organization estimates that by 2040, there will be nearly 29.5 million new cancer cases and 16.5 million cancer deaths worldwide.^1^ With growing cancer incidence and advancement in cancer treatment, demand for palliative care among patients with cancer remains high in the past decades.^2^ Palliative care refers to holistic care that enhances quality of life of patients and caregivers who face challenges associated with life‐threatening diseases.^3^ The US National Comprehensive Cancer Network (NCCN) recommends that palliative care should be offered to patients with cancer across the entire course of disease.^4^ Patients receiving cancer palliative care often experience symptoms, such as pain, fatigue, nausea and vomiting.^5^ Effectiveness of conventional pharmacological treatments for controlling these symptoms is limited, and some are associated with adverse effects.^6^

Chinese medicine is a major form of traditional, complementary and integrative medicine (TCIM), which is extensively used as an adjunctive intervention for cancer palliative care.^7^, ^8^ In Hong Kong, more than 50% of patients with cancer have consumed at least one form of Chinese medicine modalities to reduce side‐effects of conventional treatments, restrain tumour progression and improve quality of life.^9^ Chinese herbal medicine (CHM) was the most commonly used Chinese medicine modality.^9^ Findings of existing systematic reviews (SRs)^10^, ^11^ and overview of SRs^12^, ^13^, ^14^ also indicated that CHM and acupuncture and related therapies are effective in relieving pain, fatigue, constipation and improving quality of life among patients with cancer. Nonetheless, in certain areas their effectiveness remains to be uncertain due to methodological flaws among existing studies.^10^, ^11^, ^12^, ^13^, ^14^ Further clinical research should be conducted to ascertain their effectiveness.

With limited research funding for TCIM, there is an acute need in increasing research value of future clinical trials, as well as in avoiding duplication of effort and wastage of funding. Grant agencies should consider carefully what is already known or being researched currently,^15^ as well as expectations from health‐care professionals, patients and caregivers.^15^, ^16^ Prior to research priority setting, updated research evidence on Chinese medicine modalities for cancer palliative care should be systematically reviewed, and subsequently, knowledge gaps among current studies could be identified.^15^, ^17^ These gaps can then be translated to a preliminary list of clinical research questions for prioritization.

Research questions identified using the streamlined approach described above utilize existing evidence as a starting point in prioritization. This will facilitate allocation of limited research funding to confirmatory clinical trials since the priorities to be generated would have certain evidence support already. Arguably, this approach would bring the prioritization process closer to practice changes, as confirmatory trials are more likely to inform future clinical practice and guideline development.^18^

In our previous international Delphi survey,^19^ conventional physicians, Chinese medicine practitioners, nurses and clinical research methodologists prioritized eight research questions from a preliminary list generated from existing clinical evidence. However, the needs of patients with cancer, cancer survivors and caregivers in this field remain uncertain. This study aimed to prioritize top ten important Chinese medicine clinical research questions in cancer palliative care from their perspectives via a face‐to‐face prioritization workshop in Hong Kong.

## 2 METHODS

This study is approved by the Survey and Behavioural Research Ethics Committee (reference no.: 023‐17), Chinese University of Hong Kong. It comprised of three parts as follows:

### 2.1 Part 1: Preparing a list of Chinese medicine clinical research questions in cancer palliative care for prioritization

In this study, research needs are defined as existing knowledge gaps that restrict health‐care decision making by patients with cancer, cancer survivors and caregivers.^20^ Firstly, we identified research needs from two SRs^10^, ^11^ and three overviews of SRs^12^, ^13^, ^14^ on CHM and acupuncture for cancer palliative care. These syntheses were published between 2015 and 2016. They summarized up‐to‐date clinical evidence from 88 randomized controlled trials and 74 SRs at the time of our study. Secondly, we invited authors of these evidence syntheses to provide feedbacks on potential research needs. The identified potential research needs were then presented clearly using the PICO (patients, interventions, comparisons and outcomes) framework, along with the methodological limitations of existing studies (see Appendix S1). Thirdly, we converted these research needs to preliminary clinical research questions, which incorporated the PICO elements. Lastly, content of these potential research questions was amended based on comments from caregivers of patients with cancer and Chinese medicine practitioners specializing in oncology. Such amendment was to ensure that our study participants would be able to comprehend the research questions. Accordingly, a list of 25 Chinese medicine clinical research questions for cancer palliative care was prepared for prioritization in a face‐to‐face workshop (see Appendix S2).

### 2.2 Part 2: Sampling of participants

Purposive sampling strategy was used to identify a balanced number of the following three types of participants: (i) patients with cancer who have completed major cancer treatments, (ii) cancer survivors who have survived for five or more years after diagnosis and (iii) caregivers who have taken care of patients with cancer. All participants should have experiences of using Chinese medicine modalities for cancer palliative care in Hong Kong. To ensure balancing of perspectives, six participants from each of the three categories were sampled. The total number of participants was confined to 18, and this sample size has surpassed the recommended sample size for qualitative research.^21^

### 2.3 Part 3: Data collection and analysis

**FIGURE 1:**
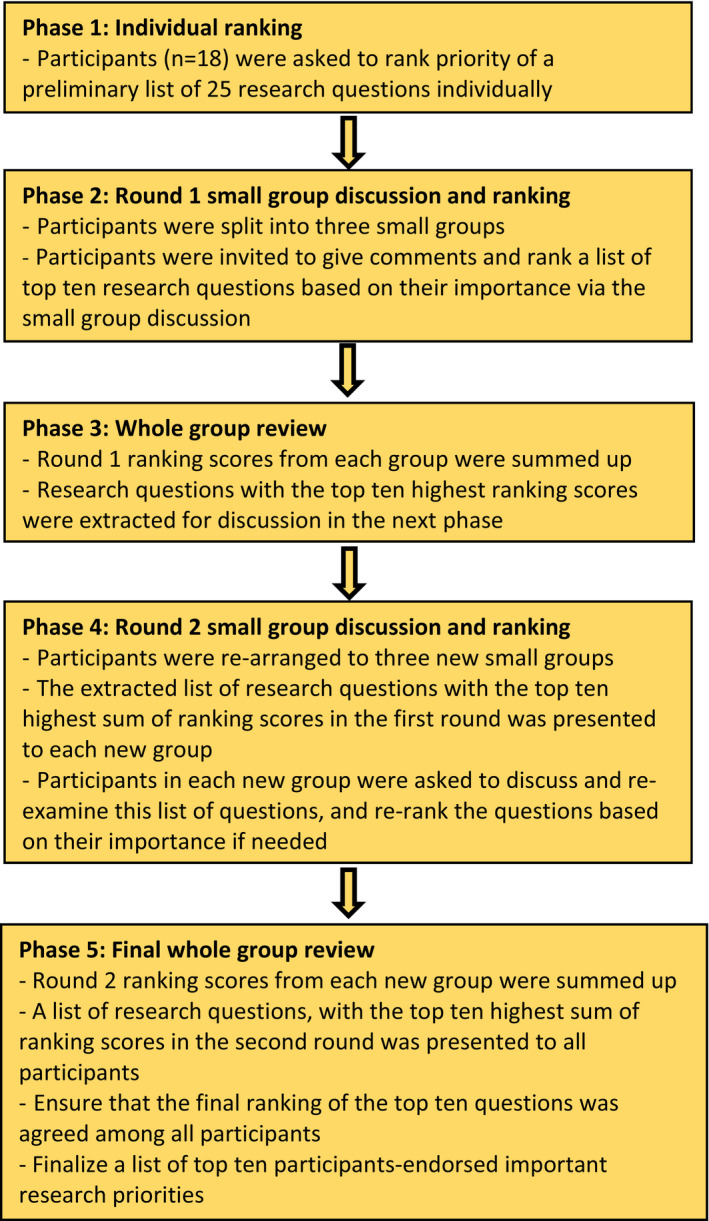
Details of the face‐to‐face workshop

Eighteen participants, including patients with cancer (n = 6), cancer survivors (n = 6) and caregivers (n = 6), were invited to a face‐to‐face prioritization workshop held on 1 June 2019. Participants were informed about the study's purpose, confidentiality of their personal information and that their participation was voluntary. Participants who agreed to join were asked to sign a written informed consent before the workshop began. Two rounds of small group discussion in the workshop were facilitated by research team members (CHLW, WW and VCHC) who were experienced in qualitative research. The workshop was comprised of five phases (Figure 1).

1. In Phase 1, participants were asked to rank the priority of the list of 25 research questions prepared in part 1 individually (see Appendix S2).
2. In Phase 2, first round of small group discussion was conducted by dividing the participants into three groups (n = 6 in each group), with a balanced number of patients with cancer, cancer survivors and caregivers (n = 2 from each category). Each participant was invited to comment on research questions which they felt were the most, and the least, important for further research. Based on the discussion, they were then asked to select top ten important research questions out of the list of 25 research questions.
3. In Phase 3, for each group, research questions on the top ten list ranking from 4th to 10th were given 1 point, while questions ranking from 1st to 3rd were given 3 points.^22^, ^23^ Then, ranking scores of all three groups in the first round were summed up.^22^, ^23^ Based on the sum of ranking scores, research questions with top ten highest consensus ranking scores were extracted for discussion in the next phase.^22^, ^23^
4. In Phase 4, second round of small group discussion was conducted by re‐arranging participants to three new groups. A list of research questions with the top ten highest sum of consensus ranking scores in the first round was presented to each new group. Question with highest consensus ranking score was ranked as 1st, while question with lowest consensus ranking scores was ranked as 10th. Participants in each new group were asked to discuss and re‐examine this list of questions, and re‐rank the questions based on their perceived importance if needed.^22^, ^23^
5. In Phase 5, data analysis similar to Phase 3 was performed. A list of research questions, with top ten highest sum of consensus ranking scores in the second round, was presented to all participants. Similar to Phase 4, the questions were arranged in a descending order, such that the question with the highest score sum were ranked as 1st. In areas where consensus was not established, agreements were reached by majority voting.^22^, ^23^ A finalized list of top ten participant‐endorsed research priorities was generated at the end of workshop.

## 3 RESULTS

### 3.1 Participants

Seventeen participants attended the face‐to‐face prioritization workshop (response rate: 94.4%), including patients with cancer who have completed major cancer treatment (n = 5), cancer survivors who have survived for five or more years after diagnosis (n = 6) and caregivers who have taken care of patients with cancer (n = 6). Of these 17 participants, 58.8% of them were female and majority of them (82.4%) were aged 40 years or above. The duration of receiving Chinese medicine modalities for cancer palliative care among patients with cancer, cancer survivors and patients with cancer who were looked after by caregivers in this study ranged from 2 months to 102 months (see Appendix S3).

### 3.2 Generation of top ten Chinese medicine clinical research priorities

From the workshop, top ten Chinese medicine clinical research priorities in cancer palliative care agreed by all participants are listed in Table 1. Details of participants’ qualitative comments on these priorities are shown in Table 2. Among these ten research priorities, five (50%) focused on acupuncture and related therapies, while five (50%) were on CHM.

**TABLE 1:** Top ten important research priorities generated from the face‐to‐face workshop

Research questions	Final ranking
Is adding manual acupuncture on top of opioids more effective in reducing pain among adult patients receiving cancer palliative care?	1
Is using CHM effective in improving quality of life among adult patients receiving cancer palliative care who undergo chemotherapy?	2
Is adding CHM on top of loperamide more effective in reducing stomatitis among adult patients receiving cancer palliative care?	3
Is adding CHM on top of conventional care (granulocyte colony‐stimulating factor (G‐CSF)/granulocyte‐macrophage colony‐stimulating factor (GM‐CSF)/interleukin‐3 (IL‐3)) more effective in improving leukopenia among adult patients receiving cancer palliative care?	4
Is adding manual acupuncture on top of opioids more effective in reducing dyspnoea among adult patients receiving cancer palliative care?	5
Is using CHM on top of progesterone analogs effective in reducing anorexia among adult patients receiving cancer palliative care who undergo chemotherapy?	6
Is adding manual acupuncture/electroacupuncture on top of neurokinin‐1 receptor antagonists + serotonin3 receptor antagonist (5‐HT3) and/or dexamethasone more effective in reducing nausea and vomiting, as well as improving quality of life among adult patients receiving cancer palliative care?	7
Is adding CHM on top of conventional care (compression bandaging and manual lymphatic drainage) more effective in reducing limbs oedema among adult patients receiving cancer palliative care?	8
Is adding CHM on top of conventional care (glycerine suppositories/lactulose syrup) more effective in reducing constipation among adult patients receiving cancer palliative care?	9
Is adding manual acupuncture on top of conventional care more effective in reducing anxiety among adult patients receiving cancer palliative care?	10

Note

The final ranking was arranged in a descending order based on the sum of ranking scores. The research question with the highest ranking scores among these top ten important research priorities was ranked as 1st.

Abbreviation: CHM, Chinese herbal medicine.

**TABLE 2:** Qualitative comments on the final list of top ten research priorities

Research questions	Final ranking	Qualitative comments
Is adding manual acupuncture on top of opioids more effective in reducing pain among adult patients receiving cancer palliative care?	1	Patient with cancer: The use of opioids usually leads to serious side‐effects. I got very drowsy and dizzy after taking opioids, then I decided not to take it instead. It would be great if manual acupuncture can help reducing the opioids dose, while retaining the pain killing effect. Cancer survivor: Pain is a very important problem among patients with cancer. Even when opioids were applied, many patients still failed to attain pain relief, and they couldn't sleep at all because of pain. We also understand that the use of opioids would bring us serious side‐effects, such as hallucinations and loss of coordination capacity. If adding manual acupuncture on top of opioids is found to be effective in pain relief among patients receiving cancer palliative care in future research, conventional physicians might consider reducing the dosage of opioids use. I think patients with cancer using opioids to relieve pain are already at their end‐of‐life stage. They are suffering from severe pain. It is extremely important to prioritize this research question because reducing pain will surely improve patients’ quality of life. Caregiver: Pain is an obstacle for patients with cancer at different stage of treatment. It not only affects physical health, but also affects psychological health. Pain is the most urgent symptom to be treated. Sometimes patients with severe pain may lose motivations to fight against cancer. I think the use of manual acupuncture should be explored in future research. Patients with cancer, cancer survivors and caregivers: Our top priority is to evaluate a potentially effective, like manual acupuncture, to reduce pain before treating other symptoms and improving quality of life among patients receiving cancer palliative care.
Is using CHM effective in improving quality of life among adult patients receiving cancer palliative care who undergo chemotherapy?	2	Patients with cancer: Quality of life is an important outcome among all patients receiving cancer palliative care. The process of communicating with CMP about my health condition, and taking the CHM prescribed by CMP helps strengthen my health and motivation in fighting against cancer. Cancer survivor: I think the importance of quality of life depends on patients’ cancer stage. Maintaining good quality of life is particularly important to patients who completed chemotherapy, of which CHM could play an important role. Patient with cancer and cancer survivor: From our personal experiences, using CHM is effective in improving quality of life. More research is needed to find out its value. Patient with cancer and caregiver: Conventional physicians recommend against the combined use of chemotherapy and CHM due to the risk of adverse herb‐drug interactions. There is a need to explore when CHM should be used in the course of cancer treatment, which would optimize the outcomes.
Is adding CHM on top of loperamide more effective in reducing stomatitis among adult patients receiving cancer palliative care?	3	Cancer survivor: When I received chemotherapy, I suffered a lot from stomatitis, and I couldn't eat anything. It is indeed important to treat stomatitis among patients receiving cancer palliative care. Without essential nutrients to nourish, repair, and heal our body, we are highly vulnerable to different health problems. The role of CHM needs urgent evaluation. Caregiver: Stomatitis not only results in appetite loss, but also brings severe pain. My mother suffered from stomatitis after undergoing chemotherapy and radiotherapy. She could not swallow anything at that time because of the severe pain caused by stomatitis. We relied on the use of CHM to reduce stomatitis and improve her overall health conditions, so I think this is an important research question. During chemotherapy, stomatitis would result in anorexia and patients cannot eat anything. As we mentioned that anorexia is undesirable. If CHM is capable of treating stomatitis, anorexia will also be reduced.
Is adding CHM on top of conventional care (granulocyte colony‐stimulating factor (G‐CSF)/granulocyte‐macrophage colony‐stimulating factor (GM‐CSF)/ interleukin‐3 (IL‐3)) more effective in improving leukopenia among adult patients receiving cancer palliative care?	4	Patient with cancer: I noticed that the use of CHM alone was effective in improving leukopenia among some patients with cancer. These patients didn't need to receive G‐CSF injection. Research is needed to prove my observation. Cancer survivor: I got mild fever and severe bone pain after G‐CSF injection. Its side‐effects made me feel even more uncomfortable than receiving chemotherapy. Hence, it is important to investigate an alternative approach like CHM to improve leukopenia. We may then reduce usage of G‐CSF injection. Even I had good appetite when receiving chemotherapy, it was still difficult to maintain white blood cell count at a normal level. I think it is very important to research the potential of CHM for improving leukopenia among patients receiving cancer palliative care, especially during or after chemotherapy. Caregiver: If the combined use of CHM and G‐CSF is effective in normalizing white blood cell level, patients with cancer may not need to delay their chemotherapy treatments. Hence, I think this research question is important.
Is adding manual acupuncture on top of opioids more effective in reducing dyspnoea among adult patients receiving cancer palliative care?	5	Patient with cancer: Dyspnoea is a very severe symptom which greatly influences our daily lives. It is hard to manage dyspnoea and we need to find out if manual acupuncture can help. Cancer survivor: I think patients with cancer under opioids treatment are already at the end‐of‐life stage. Dyspnoea problem would further worsen their quality of life. Therefore, I think it is important to investigate the effectiveness of adding manual acupuncture on top of opioids in reducing dyspnoea among these patients. Patient with cancer: When we cannot breathe well, our lung function and overall health conditions will get worse too. Thus, it is important to investigate this research question.
Is using CHM on top of progesterone analogs effective in reducing anorexia among adult patients receiving cancer palliative care who undergo chemotherapy?	6	Cancer survivor: I had poor appetite during chemotherapy and ate less than usual. There is a need to investigate CHM treatment for improving this symptom. Caregiver: Good nutrition is important for patients with cancer to nourish, repair, and heal their body. If patients with cancer cannot get essential nutrients, they may not be able to fight against cancer. Therefore, it is very important to find an approach to reduce anorexia. I notice that if patients have good appetite, they will have more motivations in fighting against cancer. For my wife's case, I think that CHM is very effective in reducing anorexia. There is a need to clarify the values of CHM so as to benefit more patients.
Is adding manual acupuncture/electroacupuncture on top of neurokinin‐1 receptor antagonists + serotonin3 receptor antagonist (5‐HT3) and/or dexamethasone more effective in reducing nausea and vomiting, as well as improving quality of life among adult patients receiving cancer palliative care?	7	Patient with cancer: Some medications for reducing nausea and vomiting are very expensive, and some patients with cancer may not be able to afford it. As the patients cannot eat anything due to these symptoms, this poses great impact on their health. Low cost options like acupuncture should be evaluated. Patient with cancer and caregiver: Nausea and vomiting demotivate patients’ fight against cancer. We should first investigate an effective approach for reducing nausea and vomiting, which would improve quality of life among patients receiving cancer palliative care greatly.
Is adding CHM on top of conventional care (compression bandaging and manual lymphatic drainage) more effective in reducing limbs oedema among adult patients receiving cancer palliative care?	8	Cancer survivor: It is saddening that patients with limbs oedema have to wear compression bandaging for the rest of their lives. Patient with cancer and cancer survivor: Limbs oedema is a very important problem to patients with cancer as there will be pain when too much fluids are trapped in the body tissues. Cancer survivor and caregiver: On top of compression bandaging and manual lymphatic drainage, I think CHM is better than acupuncture in reducing limbs oedema among adult patients receiving cancer palliative care, but this needs confirmation.
Is adding CHM on top of conventional care (glycerine suppositories/ lactulose syrup) more effective in reducing constipation among adult patients receiving cancer palliative care?	9	Cancer survivor: Constipation is one of the major side‐effects of chemotherapy and radiotherapy. After consuming CHM for a few weeks, my constipation symptoms got relieved. Caregiver: Based on my understanding, glycerine suppositories and Lactulose syrup are the most direct treatments to reduce constipation. But the additional effect offered by CHM should also be investigated, as the patient with cancer I am looking after showed improvement after using this.
Is adding manual acupuncture on top of conventional care more effective in reducing anxiety among adult patients receiving cancer palliative care?	10	Cancer survivor: As a cancer survivor, I fully understand the psychological stress after confirming the cancer diagnosis. Patients may feel anxious especially when they did not know how to relieve their symptoms. Effective Chinese medicine treatment for anxiety would therefore be very valuable as we can avoid using conventional medications, which may carry significant side‐effects. Caregiver: I think Chinese medicine clinicians performs well in managing patients’ psychological health because they are willing to provide holistic care for patients.

Abbreviations: CHM, Chinese herbal medicine; CMP, Chinese medicine practitioner.

The three most important research priorities with highest ranking scores were as follows:

1. Combining manual acupuncture and opioids for relieving pain;
2. CHM for improving quality of life among patients who undergo chemotherapy; and
3. CHM plus loperamide for reducing stomatitis.

Manual acupuncture plus opioids for relieving pain among adult patients receiving cancer palliative care was considered the most important among these three research priorities. All study participants agreed that pain reduction should be a priority before managing other symptoms or improving quality of life. One caregiver indicated that persistent pain would have significant negative impact on physical and psychological health of patients with cancer. As described by one patient with cancer and two cancer survivors, the use of opioids on pain relief might induce serious side‐effects, such as dizziness, extreme drowsiness, hallucinations and loss of coordination. Another cancer survivor suggested that conventional physicians might consider reducing the dosage of opioid use if adding manual acupuncture is found to be effective in reducing pain in future studies (Table 2).

The second‐most important research priority was the use of CHM for improving quality of life among adult patients receiving cancer palliative care who undergo chemotherapy. From their personal experiences, one patient with cancer and one cancer survivor stated that using CHM was effective in improving their quality of life, and this was considered to be an important outcome among those receiving cancer palliative care. A cancer survivor highlighted that maintaining good quality of life was essential even after the completion of chemotherapy. One patient with cancer and one caregiver expressed conventional physicians’ concerns on the potential risk of herb‐drug interactions induced by combined administration of CHM and chemotherapy (Table 2).

Concurrent use of CHM and loperamide for reducing stomatitis among adult patients receiving cancer palliative care was the third‐most important research priority. One cancer survivor mentioned that her stomatitis during chemotherapy had prevented her from feeding normally. She was worried that inadequate nutrient intake would impede her ability to recover and subsequently led to other health problems. Two caregivers also highlighted that stomatitis led to severe pain on top of appetite loss. Personal experiences of a caregiver suggested that CHM was effective in relieving stomatitis of patients with cancer, and therefore, research is needed to confirm this observation (Table 2).

### 3.3 Other qualitative comments and future research recommendations

Two caregivers indicated that research priorities should be varied for patients with cancer currently undergoing major cancer treatment, pre‐treatment and post‐treatment since the symptoms experienced by patients at different stage vary significantly. These caregivers also mentioned that symptom variations among patients with different cancer diagnoses were apparent. Therefore, separate research priority ranking based on patients’ stage of treatments and cancer diagnoses should be considered in the future (Table 2).

## 4 DISCUSSION

### 4.1 Summary of findings

In this study, patients with cancer, cancer survivors and caregivers prioritized a list of top ten Chinese medicine clinical research questions. Among them, five (50%) focused on acupuncture and related therapies, while five (50%) were on CHM. The three most important research priorities in cancer palliative care were as follows: (i) combining manual acupuncture and opioids for relieving pain; (ii) CHM for improving quality of life among patients who undergo chemotherapy; and (iii) CHM plus loperamide for reducing stomatitis.

### 4.2 Combined use of manual acupuncture and opioids for pain management as the most important research priority

Our study indicated that pain management should be prioritized among patients receiving cancer palliative care. Such finding concurred with the research direction proposed by European Society for Medical Oncology on supportive and palliative care.^24^ It is well acknowledged that cancer pain can negatively affect patients’ daily life, thus causing sleep disturbance, weakened physiological responses and increased psychosocial distress.^25^ Despite opioids being the first‐line treatment for moderate or severe cancer pain,^26^ some patients with cancer refuse to use opioids for pain management owing to fear of addiction and concerns on associated side‐effects.^27^, ^28^ Due to these considerations, it is not surprising that a recent qualitative study suggested that Chinese patients with cancer are eager to receive acupuncture for pain relief.^25^ Indeed, according to the current NCCN clinical practice guideline in oncology^29^ and the White paper issued by the US government in 2017,^30^ acupuncture is recommended for reducing cancer pain as it may reduce opioid use. To create a strong evidence base for informing appropriate co‐usage of these treatments, future research should evaluate whether combining manual acupuncture and opioids may reduce dosage of the later for pain management in cancer palliative care.

### 4.3 Concurrent use of Chinese herbal medicine and chemotherapy for improving quality of life as the second‐most important research priority

According to a population‐based survey in seven European countries, majority of the general public regarded improving quality of life as the most important priority when coping with cancer.^31^ Another qualitative study showed that most patients with cancer in the UK emphasized the importance of allocating public health‐care funding for enhancing quality of life among cancer palliative care patients.^32^ Very often chemotherapy induces various side‐effects, including but not limited to nausea, loss of appetite and fatigue. Subsequently, patients’ quality of life in both physical and psychological aspects would be worsened.^33^ Improving quality of life is therefore considered to be an important issue among patients who undergo chemotherapy.^33^ Furthermore, existing studies suggested that patients’ perceived benefits of CHM use in enhancing quality of life^34^ might outweigh their concerns on potential harms caused by herb‐drug interactions between CHM and chemotherapeutic agents.^6^ These strong patients’ preferences may explain why evaluating benefits of using CHM for improving quality of life is prioritized.

### 4.4 Use of Chinese herbal medicine plus loperamide for reducing stomatitis as the third‐most important research priority

Stomatitis could cause a decreased appetite among patients receiving cancer palliative care.^35^ Appetite reduction was considered as one of the most severe symptoms in cancer palliative care^36^, ^37^ because of its association with low nutrient intake, weight loss and weakness.^37^ Similar to other studies, caregivers in our study have placed more importance on relieving stomatitis than patients with cancer and cancer survivors themselves.^38^, ^39^ This might be due to caregivers’ belief that a good appetite would increase nutrition intake, thus improving quality of life and prolonging patients’ survival.^38^ With strong expectations from caregivers and concerns of patients with cancer on negative impacts of stomatitis, concurrent use of CHM and loperamide for this condition is also regarded as another important research priority.

### 4.5 Mismatches on perceived research priorities between experts and patients

This face‐to‐face prioritization workshop shared a similar approach with our previous international Delphi survey^19^ on identifying the list of preliminary research questions. The research questions in both studies were developed based on knowledge gaps generated from the same body of evidence.^10^, ^11^, ^12^, ^13^, ^14^ In the international Delphi survey, eight Chinese medicine clinical research questions for cancer palliative care were prioritized by conventional physicians, Chinese medicine practitioners, nurses and clinical research methodologists.^19^ It is noteworthy that research questions prioritized by health‐care professionals might not be aligned to those experiencing the disease.^40^ We hence compared these eight international expert‐endorsed priorities^19^ with the ten research questions prioritized among patients and caregivers in our workshop (see Appendix S4).

International experts’ choices^19^ did not align with local preference on second‐most and third‐most important research priorities (see Appendix S4). A possible reason for such mismatches was that experts and patients held different perceptions towards CHM safety and negative herb‐drug interaction.^6^, ^19^ While experts expressed concerns over these safety issues,^19^ patients with cancer regarded symptom alleviation from CHM use would outweigh the risk of adverse events.^6^ Health‐care professionals’ communications with patients and caregivers regarding rational use of CHM are thus important.^6^, ^41^ However, current evidence based on safety of CHM and herb‐drug interactions in cancer palliative care is very limited. Observational and pharmacological studies should be conducted to evaluate possible harm prior to performing relevant clinical trials.^42^

Combining manual acupuncture and opioids for pain management among patients receiving cancer palliative care was the most important prioritized question among international experts^19^ and local patients (see Appendix S4). The other two co‐prioritized research questions were CHM plus conventional care for reducing constipation (i.e. the ninth‐most important in the current workshop), as well as manual acupuncture plus conventional care for reducing anxiety (i.e. the tenth‐most important in the current workshop). Grant agencies might consider addressing these three co‐prioritized questions as priorities.

### 4.6 Strengths and limitations of this study

In this workshop, patients and caregivers from diverse background could express their expectations via structured interactions. Through ranking exercise within the small group discussions, every participant's viewpoints could be considered.^22^, ^23^ The domination of discussion by a single participant was also prevented.^22^, ^23^ Besides, patients and caregivers could feel empowered by making contributions to prioritizing research questions.^43^

Future research needs may be identified from knowledge gaps on other Chinese medicine interventions, such as tai chi^44^ and cupping therapy.^45^ Our research priorities were generated among local participants who have experiences of using Chinese medicine modalities for cancer palliative care. Hence, findings might not be directly generalizable to other health system context and culture. Our study participants were not categorized according to patients' stage of treatments and cancer diagnoses. Nevertheless, our findings were agreed by all participants. These prioritized research questions remain to be highly relevant regardless of cancer stage and severity.

## 5 CONCLUSION

We prioritized a list of top ten important Chinese medicine clinical research questions for cancer palliative care among patients with cancer, cancer survivors and caregivers in Hong Kong. These priorities will guide clinical researchers on future direction and will inform grant agencies on rational allocation of limited funding. To maximize research value and minimize wastage of funding, it is important to align the priorities from both professional and patient perspectives. The most important co‐prioritized research question may be addressed first with appropriate support in the near future.

## CONFLICT OF INTEREST

None.

## AUTHORS' CONTRIBUTIONS

Charlene HL Wong and Vincent CH Chung contributed to conception and design. Charlene HL Wong, Wendy Wong and Vincent CH Chung performed acquisition of data, analysis and interpretation of data and statistical analysis. Charlene HL Wong drafted the manuscript. Wendy Wong, Justin CY Wu, Ting Hung Leung, Irene XY Wu and Vincent CH Chung critically revised the manuscript for important intellectual content. Vincent CH Chung obtained funding. Wendy Wong, Wai Ling Lin and David KY Au gave administrative, technical or material support. Vincent CH Chung underwent supervision.

## Supporting information

Appendices S1‐S4

## Data Availability

The data that support the findings of this study are available on request from the corresponding author. The data are not publicly available due to privacy or ethical restrictions.
