# Supplementary material for: Prioritizing Chinese medicine clinical research questions in cancer palliative care from patient and caregiver perspectives
Source: Health Expect. 2021 Jun 9;24(4):1487–97. doi: 10.1111/hex.13289 (PMC8369121; doi:10.1111/hex.13289)
Supplement: Supplementary file 1 — Appendices S1‐S4 [file HEX-24-1487-s001.docx]

Appendix 1. Potential research needs of Chinese medicine modalities for cancer palliative care identified from existing SRs and overview of SRs [1-5] and authors of these studies

| No. | Methodological limitations | Population | Intervention | Comparison | Outcomes |
| --- | --- | --- | --- | --- | --- |
| 1 | Small sample size and High risk of bias | Palliative cancer patients with nausea and vomiting | Electroacupuncture plus Neurokinin-1 receptor antagonists + serotonin_3_ receptor antagonist (5-HT_3_) and/or Dexamethasone | Neurokinin-1 receptor antagonists + Serotonin_3_ receptor antagonist (5-HT_3_) and/or Dexamethasone alone | Nausea and vomiting, quality of life |
| 2 | Small sample size,  High risk of bias and Conflicting results | Palliative cancer patients with nausea and vomiting | Manual acupuncture plus Neurokinin-1 receptor antagonists + serotonin_3_ receptor antagonist (5-HT_3_) and/or Dexamethasone | Neurokinin-1 receptor antagonists + Serotonin_3_ receptor antagonist (5-HT_3_) and/or Dexamethasone alone | Nausea and vomiting, quality of life |
| 3 | Small sample size and High risk of bias | Palliative cancer patients with paresthesia and dysesthesia | Electroacupuncture plus Vitamin B6 (pyridoxine) supplement | Vitamin B6 (pyridoxine) supplement alone | Paresthesia and dysesthesia |
| 4 | Small sample size,  High risk of bias and Conflicting results | Palliative cancer patients with fatigue | Manual acupuncture plus Exercise | Exercise alone | Fatigue |
| 5 | Conflicting results and High risk of bias | Palliative cancer patients with fatigue | Moxibustion plus Exercise | Exercise alone | Fatigue |
| 6 | Small sample size,  High risk of bias and Conflicting results | Palliative cancer patients with anorexia | Manual acupuncture plus Orexigenic agents^ | Orexigenic agents^ alone | Anorexia |
|  | Note: ^ In Canada, practitioners are able to select from a variety of pharmaceutical agents: Corticosteriod (short-term) / Progestinal agents (e.g., megestrol acetate) / Metoclopramide (for nausea and vomiting) / Omega 3 (less common) / Dronabinal (less common) | | | | |
| 7 | Small sample size and High risk of bias | Palliative cancer patients with anorexia | Qi-ge-kai-wei decoction (啟膈開胃湯) plus Orexigenic agents^ | Orexigenic agents^ alone | Anorexia |
|  | Note: ^ In Canada, practitioners are able to select from a variety of pharmaceutical agents: Corticosteriod (short-term) / Progestinal agents (e.g., megestrol acetate) / Metoclopramide (for nausea and vomiting) / Omega 3 (less common) / Dronabinal (less common)  Details of Qi-ge-kai-wei decoction (啟膈開胃湯): Astragalus mongholicus Bunge [Sheng Huang Qi生黃芪] 30g, Poria [Fu Ling茯苓] 30g, Coix lacryma-jobi L. var. mayuen (Roman.) Stapf [Mi Ren 米仁] 30g, Mixed Amomi Fructus and Amomi Fructus Rotundus [Sha Kou Ren砂蔻仁]5g, Atractylodes macrocephala Koidz. [Chao Bai Zhu 炒白術] 15g, Bambusa tuldoides Munro [Jiang Zhu Ru姜竹茹] 10g, Perilla frutescens (L.) Britt. [Su Geng 蘇梗] 10g | | | | |
| 8 | Small sample size and High risk of bias | Palliative cancer patients with anorexia | Tong-tai decoction (通泰合劑) plus Chemotherapy + progesterone analogs | Chemotherapy + progesterone analogs | Anorexia |
|  | Note: Details of Tong-tai mixture (通泰合劑): Astragalus mongholicus Bunge [Sheng Huang Qi生黃芪], Coix lacryma-jobi L. var. mayuen (Roman.) Stapf [Yi Yi Ren薏苡仁], Curcuma phaeocaulis Valeton [E Zhu莪術], Spatholobus suberectus Dunn [Ji Xue Teng, 雞血藤], Sinapis alba L. [Bai Jie Zi白芥子], Patrinia rupestris [Mu Tou Hui墓頭回], Agrimonia pilosa Ledeb. [Xian He Cao仙鶴草], Scleromitrion diffusum(Willd.) R.J.Wang[Bai Hua She She Cao白花蛇舌草], Atractylodes macrocephala Koidz. [Bai Zhu白術]. | | | | |
| 9 | Small sample size,  High risk of bias and Conflicting results | Palliative cancer patients with pain | Manual acupuncture plus Opioids | Opioids alone | Pain |
| 10 | Small sample size and High risk of bias | Palliative cancer patients with pain | Transcutaneous electrical nerve stimulation (TENS) | Sham TENS | Pain |
| 11 | Small sample size,  High risk of bias and Conflicting results | Palliative cancer patients with hot flashes | Manual acupuncture plus Conventional care (SSRI (antidepressants)/ clonidine/Effexor/ gabapentin/hormone therapy/ paroxetine(brisdelle)) | Conventional care  (SSRI (antidepressants)/ clonidine/Effexor/ gabapentin/hormone therapy/ paroxetine(brisdelle)) | Hot flashes |
| 12 | Conflicting results | Palliative cancer patients with hot flashes | Manual acupuncture | Sham acupuncture | Hot flashes and quality of life |
| 13 | Small sample size | Palliative cancer patients | Moxibustion plus Wei-chang-an (胃腸安) | Wei-chang-an (胃腸安) alone | Quality of life |
|  | Note: Details of Wei-chang-an (胃腸安): Pseudostellaria heterophylla (Miq.) Pax [Tai Zi Shen太子參] 12g, Atractylodes macrocephala Koidz. [Bai Zhu白術]12g, Poria [Fu Ling茯苓] 30g, Sargentodoxa cuneata (Oliv.) Rehder & E.H.Wilson [Hong Teng紅藤] 30g, Prunella vulgaris L. [Xia Ku Cao夏枯草] 9g, Smilax china L. [Ba Qia菝葜] 30g, [Lv E Mei綠萼梅] 9g, etc | | | | |
| 14 | Small sample size and Short follow up duration (1 week only) | Palliative cancer patients | Chinese herbal medicine  (e.g. Yi-fei-bai-du decoction (益肺敗毒湯), Fei-liu-ping extract (肺瘤平膏), Hai-shen-su (海參素) and Fu-zheng-jie-du decoction (扶正解毒湯)) plus Chemotherapy | Chemotherapy alone | Quality of life |
|  | Note: Details of Chinese herbal medicine:   1. Yi-fei-bai-du decoction(益肺敗毒湯): Panax quinquefolius L. [Bai Shen白參(蒸兌)] 10g, Astragalus mongholicus Bunge [Sheng Huang Qi生黃芪] 30g, Ganoderma lucidum [Ling Zhi靈芝] 30g, Adenophora triphylla (Thunb.) A.DC. [Sha Shen沙參] 12g, Ophiopogon japonicus (Thunb.) Ker Gawl. [Mai Dong麥冬] 12g, Lilium lancifolium Thunb. [Bai He百合] 15g, Rehmannia glutinosa (Gaertn.) DC. [Sheng Di生地] 12g, Fritillaria thunbergii Miq. [ Zhe Bei Mu浙貝母] 10g, Platycodon grandiflorus (Jacq.) A.DC. [Jue Geng橘梗] 10g, Salvia miltiorrhiza Bunge [Dan Shen丹參] 15g, Clerodendrum bungei Steud. [Chou Mu Dan臭牡丹] 30g, [Shi Jian Chuan石見穿] 30g, Scleromitrion diffusum (Willd.) R.J.Wang [Bai Hua She She Cao白花蛇舌草] 30g, Trichosanthes kirilowii Maxim. [Gua Lou瓜蔞] 10g, Glycyrrhiza uralensis Fisch. ex DC. [Gan Cao甘草] 6g. 2. Fei-liu-ping extract (肺瘤平膏)：原肺瘤平二號膏，Astragalus mongholicus Bunge [Huang Qi黃芪], Panax quinquefolius L. [Xi Yang Shen西洋參]、Adenophora triphylla (Thunb.) A.DC. [Sha Shen沙參]、Ophiopogon japonicus (Thunb.) Ker Gawl. [Mai Dong麥冬]、Prunus persica (L.) Batsch [Tao Ren桃仁]、Panax notoginseng (Burkill) F.H.Chen [San Qi三七]、Scleromitrion diffusum (Willd.) R.J.Wang [Bai Hua She She Cao白花蛇舌草]、Bistorta officinalis Delarbre [Quan Shen拳參]、Patrinia scabiosifolia Link [Bai Jiang Cao敗醬草]、Paris polyphylla Sm. [Cao He Che草河車], etc., produced by Guang'anmen Hospital of China Academy of Chinese Medical Sciences，Manufacturing code京藥製字Z20063236，250g/bottle，1.63g/ capsule. 3. Hai-shen-su (海參素)：An extract from Tegillarca granosa (major chemical constituents are albumen with a molecular weight ranged from 15 KDa to 23 KDa) 4. Fu-zheng-jie-du decoction (扶正解毒湯): Panax ginseng C. A. Mey. [Ren Shen人參], Atractylodes macrocephala Koidz.[Bai Zhu白術], Poria [Fu Ling茯苓], Glycyrrhiza uralensis Fisch. ex DC. [Gan Cao甘草], Astragalus mongholicus Bunge [Huang Qi黃芪], Angelica sinensis (Oliv.) Diels [Dang Gui當歸], Spatholobus suberectus Dunn [Ji Xue Teng雞血藤], Pyrrosia lingua (Thunb.) Farw. [Shi Wei石葦], Scutellaria barbata D.Don [Ban Zhi Lian半枝蓮], Cyperus rotundus L. [Xiang Fu香附], Prunus persica (L.) Batsch [Tao Ren桃仁], arthamus tinctorius L.[Hong Hua紅花], etc. | | | | |
| 15 | Small sample size | Palliative cancer patients with xerostomia | Manual acupuncture plus Artificial saliva | Artificial saliva alone | Xerostomia |
| 16 | Small sample size | Palliative cancer patients with dyspnea | Manual acupuncture plus Opioids | Opioids alone | Dyspnea |
| 17 | Small sample size | Palliative cancer patients with lymphedema | Manual acupuncture plus Manual lymphatic drainage | Manual lymphatic drainage alone | Lymphedema |
| 18 | Small sample size | Palliative cancer patients | Acupressure | Sham acupressure | Insomnia |
| **Below are the research needs that no specific Chinese herbal medicine was provided by available studies. The co-authors did not provide any specific recommendations for the Chinese herbal medicine as well.** | | | | | |
| 19 | Conflicting results | Palliative cancer patients | Chinese herbal medicine plus Conventional care  *(as recommended by co-authors: compression banadaging/ manual lymphatic drainage)* | Conventional care  *(as recommended by co-authors: compression banadaging/ manual lymphatic drainage)* | Limbs edema |
| 20 | Conflicting results | Palliative cancer patients | Chinese herbal medicine plus Conventional care  *(as recommended by co-authors: Glycerin suppositories/ Lactulose syrup)* | Conventional care  *(as recommended by co-authors: Glycerin suppositories/ Lactulose syrup)* | Constipation |
| 21 | Conflicting results | Palliative cancer patients | Chinese herbal medicine plus Conventional care  *(as recommended by co-authors: Granulocyte-colony stimulating factor (G-CSF)/ granulocytemacrophage colony stimulating factor (GM-CSF)/ interleukin 3 (IL3))* | Conventional care  *(as recommended by co-authors: Granulocyte-colony stimulating factor (G-CSF)/ granulocytemacrophage colony stimulating factor (GM-CSF)/ interleukin 3 (IL3))* | Leukopenia |
| 22 | Conflicting results | Palliative cancer patients | Chinese herbal medicine plus Conventional care  *(as recommended by co-authors: interleukin 3 (IL3)/ platelet transfusion)* | Conventional care  *(as recommended by co-authors: interleukin 3 (IL3)/ platelet transfusion)* | Thrombocytopenia |
| 23 | Conflicting results | Palliative cancer patients | Chinese herbal medicine plus Conventional care  *(as recommended by co-authors: blood transfusion/ erythropoiesis-stimulating agents (e.g., epoetin))* | Conventional care  *(as recommended by co-authors: blood transfusion/ erythropoiesis-stimulating agents (e.g., epoetin))* | Anemia |
| 24 | Conflicting results | Palliative cancer patients | Chinese herbal medicine plus Loperamide | Loperamide alone | Diarrhea and stomatitis |
| 25 | Recommended by co-authors | Palliative cancer patients with malignant brain tumor | Chinese herbal medicine + Conventional care | Conventional care alone | Brain function, cognitive performance and memory loss |

Key: SRs, systematic reviews.

References of Appendix 1

[1] V.C. Chung, X.Y. Wu, E.P. Hui, E.T. Ziea, B.F. Ng, R.S. Ho, K.K. Tsoi, S.Y. Wong, J.C. Wu, Effectiveness of Chinese herbal medicine for cancer palliative care: overview of systematic reviews with meta-analysis, Scientific Reports (5) (2015) 18111.

[2] X.Y. Wu, V.C.H. Chung, E.P. Hui, E.T.C. Ziea, B.F.L. Ng, R.S.T. Ho, K.K.F. Tsoi, S.Y.S. Wong, J.C.Y. Wu, Effectiveness of acupuncture and related therapies for palliative care of cancer: overview of systematic reviews, Scientific Reports 5 (2015) 16776.

[3] X.Y. Wu, V.C.H. Chung, P. Lu, S.K. Poon, E.P. Hui, A.Y.L. Lau, L.G. Balneaves, S.Y.S. Wong, J.C.Y. Wu, Chinese Herbal Medicine for Improving Quality of Life Among Nonsmall Cell Lung Cancer Patients: Overview of Systematic Reviews and Network Meta-Analysis, Medicine 95(1) (2016) e2410.

[4] C. Lau, X. Wu, V. Chung, X. Liu, E. Hui, H. Cramer, R. Lauche, S. Wong, A. Lau, R. Sit, E. Ziea, B. Ng, J. Wu, Acupuncture and related therapiesy for symptom management in palliative cancer care: systematic review and meta-analysis, Medicine (Baltimore) 95(9) (2016) e2901.

[5] V. Chung, X. Wu, P. Lu, E. Hui, Y. Zhang, A. Zhang, A. Lau, J. Zhao, M. Fan, E. Ziea, B. Ng, S. Wong, J. Wu, Chinese herbal medicine for symptom management in cancer palliative care: systematic review and meta-analysis, Medicine (Baltimore) 95(7) (2016) e2793.

Appendix 2. List of research questions presented at the beginning of the face-to-face workshop

| Research questions |
| --- |
| Intervention: Acupuncture and related therapies |
| 1. Is adding Manual Acupuncture on top of opioids more effective in reducing pain among adult patients receiving cancer palliative care? |
| 1. Is adding Manual Acupuncture on top of opioids more effective in reducing dyspnea among adult patients receiving cancer palliative care? |
| 1. Is adding Manual Acupuncture/ Moxibustion on top of exercise more effective in reducing fatigue among adult patients receiving cancer palliative care? |
| 1. Is adding Manual Acupuncture on top of conventional care more effective in reducing anxiety among adult patients receiving cancer palliative care? |
| 1. Is adding Manual Acupuncture on top of orexigenic agents^ more effective in reducing anorexia among adult patients receiving cancer palliative care?   Note: ^In Canada, practitioners are able to select from a variety of pharmaceutical agents: Corticosteriod (short-term)/ Progestinal agents (e.g. megestrol acetate)/ Metoclopramide (for nausea and vomiting)/ Omega 3 (less common)/ Dronabinal (less common) |
| 1. Is adding Manual Acupuncture/ Electroacupuncture on top of Neurokinin-1 receptor antagonists + serotonin3 receptor antagonist (5-HT3) and/or Dexamethasone more effective in reducing nausea and vomiting, as well as improving quality of life among adult patients receiving cancer palliative care? |
| 1. Is adding Manual Acupuncture on top of conventional care (SSRI (antidepressants)/ clonidine/Effexor/ gabapentin/hormone therapy/ paroxetine (brisdelle)) more effective in reducing hot flashes among adult patients receiving cancer palliative care? |
| 1. Is adding Manual Acupuncture on top of manual lymphatic drainage more effective in reducing lymphedema among adult patients receiving cancer palliative care? |
| 1. Is adding Electroacupuncture on top of oral vitamin B6 (pyridoxine) supplement more effective in reducing paresthesia and dysesthesia among adult patients receiving cancer palliative care? |
| 1. Is adding Manual Acupuncture on top of artificial saliva more effective in reducing xerostomia among adult patients receiving cancer palliative care? |
| 1. Is Manual Acupuncture more effective than sham acupuncture in improving quality of life and reducing hot flashes among adult patients receiving cancer palliative care? |
| 1. Is Transcutaneous Electrical Nerve Stimulation (TENS) more effective than sham TENS in reducing pain among adult patients receiving cancer palliative care? |
| 1. Is acupressure provided by Chinese medicine practitioners more effective than self-acupressure in improving insomnia among adult patients receiving cancer palliative care and chemotherapy? |
| Intervention: Chinese herbal medicine (CHM) |
| 1. Is adding CHM on top of conventional care (Glycerin suppositories/ Lactulose syrup) more effective in reducing constipation among adult patients receiving cancer palliative care?   Note: Details of CHM:  a) Panax ginseng C. A. Mey. [Ren Shen人參], Astragalus mongholicus Bunge [Huang Qi黃茋], Atractylodes macrocephala Koidz. [Bai Zhu白術], Magnolia officinalis Rehder & E.H.Wilson [Cortex Magnoliae Officinalis厚朴], Citrus aurantium L. [Zhi shi枳實], Rheum palmatum L. [Da huang大黃], Paeonia lactiflora Pall. [Bai Shao白芍], Codonopsis pilosula (Franch.) Nannf. [Dang Shen黨參], Cornus officinalis Siebold & Zucc. [Shan Zhu Yu山茱萸];  b) Chengqi decoction [承氣湯類], Apricot Seed & Linum Formula [Ma Zi Ren Wan麻子仁丸] |
| 1. Is adding CHM on top of orexigenic agents^ more effective in reducing anorexia among adult patients receiving cancer palliative care?   Note: Details of CHM:  Qi-ge-kai-wei decoction (啟膈開胃湯): Astragalus mongholicus Bunge [Sheng Huang Qi生黃芪] 30g, Poria [Fu Ling茯苓] 30g, Coix lacryma-jobi L. var. mayuen (Roman.) Stapf [Mi Ren 米仁] 30g, Mixed Amomi Fructus and Amomi Fructus Rotundus [Sha Kou Ren砂蔻仁]5g, Atractylodes macrocephala Koidz. [Chao Bai Zhu 炒白術] 15g, Bambusa tuldoides Munro [Jiang Zhu Ru姜竹茹] 10g, Perilla frutescens (L.) Britt. [Su Geng 蘇梗] 10g  ^In Canada, practitioners are able to select from a variety of pharmaceutical agents: Corticosteriod (short-term)/ Progestinal agents (e.g. megestrol acetate)/ Metoclopramide (for nausea and vomiting)/ Omega 3 (less common)/ Dronabinal (less common) |
| 1. Is adding CHM on top of conventional care (Blood transfusion/ Erythropoiesis-stimulating agents (e.g. Epoetin)) more effective in improving anemia among adult patients receiving cancer palliative care?   Note: Details of CHM:  a) Adjusted Angelicae Sinensis Decoction for Supplementing Blood [當歸補血湯加減];  b) Adjusted Shiquan Dabu Decoction [十全大補湯加減 (Astragalus mongholicus Bunge [Huang Qi黃茋]), Panax ginseng C. A. Mey. [Ren Shen人參], Angelica sinensis (Oliv.) Diels [Dang Gui當歸], Paeonia lactiflora Pall. [Bai Shao白芍], Atractylodes macrocephala Koidz. [Bai Zhu白術], Cibotium barometz (L.) J.Sm. [Gou Qi枸杞], Colla Corii Asini [E Jiao阿膠], Glycyrrhiza uralensis Fisch. ex DC. [Zhi Gan Cao炙甘草], Salvia miltiorrhiza Bunge [Dan Shen丹參], Panax notoginseng (Burkill) F.H.Chen [San Qi三七])] |
| 1. Is adding CHM on top of conventional care (Granulocyte-colony stimulating factor (G-CSF)/ granulocytemacrophage colony stimulating factor (GM-CSF)/ interleukin 3 (IL3)) more effective in improving leukopenia among adult patients receiving cancer palliative care?   Note: Details of CHM:  a) Adjusted Angelicae Sinensis Decoction for Supplementing Blood [當歸補血湯加減];  b) Adjusted Shiquan Dabu Decoction [十全大補湯加減(Astragalus mongholicus Bunge [Huang Qi黃茋]), Panax ginseng C. A. Mey. [Ren Shen人參], Angelica sinensis (Oliv.) Diels [Dang Gui當歸], Paeonia lactiflora Pall. [Bai Shao白芍], Atractylodes macrocephala Koidz. [Bai Zhu白術], Cibotium barometz (L.) J.Sm. [Gou Qi枸杞], Colla Corii Asini [E Jiao阿膠], Glycyrrhiza uralensis Fisch. ex DC. [Zhi Gan Cao炙甘草], Salvia miltiorrhiza Bunge [Dan Shen丹參], Panax notoginseng (Burkill) F.H.Chen [San Qi三七])];  c) Adjusted Bazhen Decoction [八珍湯加減] |
| 1. Is adding CHM on top of conventional care (interleukin 3 (IL3)/ platelet transfusion) more effective in improving thrombocytopenia among adult patients receiving cancer palliative care?   Note: Details of CHM:  a) Adjusted Angelicae Sinensis Decoction for Supplementing Blood [當歸補血湯加減];  b) Adjusted Shiquan Dabu Decoction [十全大補湯加減 (Astragalus mongholicus Bunge [Huang Qi黃芪], Panax ginseng C. A. Mey. [Ren Shen人參], Angelica sinensis (Oliv.) Diels [Dang Gui當歸], Paeonia lactiflora Pall. [Bai Shao白芍], Atractylodes macrocephala Koidz. [Bai Zhu白術], Cibotium barometz (L.) J.Sm. [Gou Qi枸杞], Colla Corii Asini [E Jiao阿膠], Glycyrrhiza uralensis Fisch. ex DC. [Zhi Gan Cao炙甘草], Salvia miltiorrhiza Bunge [Dan Shen丹參], Panax notoginseng (Burkill) F.H.Chen [San Qi三七])];  c) Arachis hypogaea L. [Peanut skin 花生皮] |
| 1. Is adding CHM on top of conventional care (compression bandaging and manual lymphatic drainage) more effective in reducing limbs edema among adult patients receiving cancer palliative care?   Note: Details of CHM:  a) For the previous case: Liquidambar formosana Hance [Lu Lu Tong 路路通], Curcuma phaeocaulis Valeton [E Shu 莪術], Forsythia suspensa (Thunb.) Vahl [Lian Qiao 連翹], Smilax glabra Roxb. [Tu Fu Ling 土茯苓], Coix lacryma-jobi L. var. mayuen (Roman.) Stapf [Yi Ren 苡仁], Poria [Fu Ling茯苓], Tetrapanax papyrifer (Hook.) K.Koch [Tong Cao 通草]; b) For later case: Descurainia sophia (L.) Webb ex Prantl [Ting Li Zi 葶藶子], Plantago asiatica L. [Che Qian Zi 車前子], Ziziphus jujuba Mill. [Da Zao 大棗], Astragalus mongholicus Bunge [Huang Qi黃茋], Panax ginseng C. A. Mey. [Ren Shen人參], Angelica sinensis (Oliv.) Diels [Dang Gui當歸], Paeonia lactiflora Pall. [Bai Shao白芍], Atractylodes macrocephala Koidz. [Bai Zhu白術];  c) Wu Ling San五苓散, Adjusted Zhu Ling Decoction豬苓湯加減 |
| 1. Is adding CHM on top of loperamide more effective in reducing diarrhea among adult patients receiving cancer palliative care?   Note: Details of CHM:  a) Adjusted Tonify the Middle & Augment the Qi Decoction [補中益氣湯加減 (Actaea heracleifolia (Kom.) J.Compton [Sheng Ma升麻], Mentha canadensis L. [Bo He薄荷], Panax ginseng C. A. Mey. [Ren Shen人參], Astragalus mongholicus Bunge [Huang Qi黃茋], Atractylodes macrocephala Koidz. [Bai Zhu白術], Codonopsis pilosula (Franch.) Nannf. [Dang Shen黨參], Saposhnikovia divaricata (Turcz.) Schischk. [Fang Feng防風], Dipsacus inermis Wall. [Xu Duan續斷], Eucommia ulmoides Oliv. [Du Zhong杜仲], Rosa laevigata Michx. [Jin Ying Zi金櫻子], Euryale ferox Salisb. [Qian Shi芡實] |
| 1. Is adding CHM on top of loperamide more effective in reducing stomatitis among adult patients receiving cancer palliative care?   Note: Details of CHM:  a) Adjusted Tonify the Middle & Augment the Qi Decoction [補中益氣湯加減 (Actaea heracleifolia (Kom.) J.Compton [Sheng Ma升麻], Mentha canadensis L. [Bo He薄荷], Panax ginseng C. A. Mey. [Ren Shen人參], Astragalus mongholicus Bunge [Huang Qi黃茋], Atractylodes macrocephala Koidz. [Bai Zhu白術], Codonopsis pilosula (Franch.) Nannf. [Dang Shen黨參], Saposhnikovia divaricata (Turcz.) Schischk. [Fang Feng防風], Dipsacus inermis Wall. [Xu Duan續斷], Eucommia ulmoides Oliv. [Du Zhong杜仲], Rosa laevigata Michx. [Jin Ying Zi金櫻子], Euryale ferox Salisb. [Qian Shi芡實] |
| 1. Is using CHM effective in improving quality of life among adult patients receiving cancer palliative care and chemotherapy?   Note: Details of CHM:   1. Yi-fei-bai-du decoction(益肺敗毒湯): Panax quinquefolius L. [Bai Shen白參(蒸兌)] 10g, Astragalus mongholicus Bunge [Sheng Huang Qi生黃芪] 30g, Ganoderma lucidum [Ling Zhi靈芝] 30g, Adenophora triphylla (Thunb.) A.DC. [Sha Shen沙參] 12g, Ophiopogon japonicus (Thunb.) Ker Gawl. [Mai Dong麥冬] 12g, Lilium lancifolium Thunb. [Bai He百合] 15g, Rehmannia glutinosa (Gaertn.) DC. [Sheng Di生地] 12g, Fritillaria thunbergii Miq. [ Zhe Bei Mu浙貝母] 10g, Platycodon grandiflorus (Jacq.) A.DC. [Jue Geng橘梗] 10g, Salvia miltiorrhiza Bunge [Dan Shen丹參] 15g, Clerodendrum bungei Steud. [Chou Mu Dan臭牡丹] 30g, [Shi Jian Chuan石見穿] 30g, Scleromitrion diffusum (Willd.) R.J.Wang [Bai Hua She She Cao白花蛇舌草] 30g, Trichosanthes kirilowii Maxim. [Gua Lou瓜蔞] 10g, Glycyrrhiza uralensis Fisch. ex DC. [Gan Cao甘草] 6g. 2. Fei-liu-ping extract (肺瘤平膏)：原肺瘤平二號膏，Astragalus mongholicus Bunge [Huang Qi黃芪], Panax quinquefolius L. [Xi Yang Shen西洋參]、Adenophora triphylla (Thunb.) A.DC. [Sha Shen沙參]、Ophiopogon japonicus (Thunb.) Ker Gawl. [Mai Dong麥冬]、Prunus persica (L.) Batsch [Tao Ren桃仁]、Panax notoginseng (Burkill) F.H.Chen [San Qi三七]、Scleromitrion diffusum (Willd.) R.J.Wang [Bai Hua She She Cao白花蛇舌草]、Bistorta officinalis Delarbre [Quan Shen拳參]、Patrinia scabiosifolia Link [Bai Jiang Cao敗醬草]、Paris polyphylla Sm. [Cao He Che草河車], etc., produced by Guang'anmen Hospital of China Academy of Chinese Medical Sciences，Manufacturing code京藥製字Z20063236，250g/bottle，1.63g/ capsule. 3. Hai-shen-su (海參素)：An extract from Tegillarca granosa (major chemical constituents are albumen with a molecular weight ranged from 15 KDa to 23 KDa) 4. Fu-zheng-jie-du decoction (扶正解毒湯): Panax ginseng C. A. Mey. [Ren Shen人參], Atractylodes macrocephala Koidz.[Bai Zhu白術], Poria [Fu Ling茯苓], Glycyrrhiza uralensis Fisch. ex DC. [Gan Cao甘草], Astragalus mongholicus Bunge [Huang Qi黃芪], Angelica sinensis (Oliv.) Diels [Dang Gui當歸], Spatholobus suberectus Dunn [Ji Xue Teng雞血藤], Pyrrosia lingua (Thunb.) Farw. [Shi Wei石葦], Scutellaria barbata D.Don [Ban Zhi Lian半枝蓮], Cyperus rotundus L. [Xiang Fu香附], Prunus persica (L.) Batsch [Tao Ren桃仁], arthamus tinctorius L.[Hong Hua紅花], etc. |
| 23) Is using CHM on top of progesterone analogs effective in reducing anorexia among adult patients receiving cancer palliative care and chemotherapy?  Note: Details of CHM:   1. Tong-tai mixture (通泰合劑): Astragalus mongholicus Bunge [Sheng Huang Qi生黃芪], Coix lacryma-jobi L. var. mayuen (Roman.) Stapf [Yi Yi Ren薏苡仁], Curcuma phaeocaulis Valeton [E Zhu莪術], Spatholobus suberectus Dunn [Ji Xue Teng, 雞血藤], Sinapis alba L. [Bai Jie Zi白芥子], Patrinia rupestris [Mu Tou Hui墓頭回], Agrimonia pilosa Ledeb. [Xian He Cao仙鶴草], Scleromitrion diffusum (Willd.) R.J.Wang [Bai Hua She She Cao白花蛇舌草], Atractylodes macrocephala Koidz. [Bai Zhu白術]. |
| 24) Is adding CHM on top of conventional care more effective in improving brain function, cognitive performance and memory loss among patients receiving cancer palliative care and with malignant brain tumours?  Note: Details of CHM:  a) [Shi Jian Chuan石見穿], Acorus calamus var. angustatus Besser [Shi Chang Pu石菖蒲], Gypsophila vaccaria (L.) Sm. [Wang Bu Liu Xing王不留行], Trionycis Carapax [Bie Jia鱉甲], Anemarrhena asphodeloides Bunge [Zhi Mu知母], Salvia miltiorrhiza Bunge [Dan Shen丹參], Smilax glabra Roxb. [Tu Fu Ling 土茯苓], Cibotium barometz (L.) J.Sm. [Gou Qi枸杞];  b) Orifices opening and insect drug [Kai Qiao Yao and Chong Lei Yao開竅及蟲類藥] |
| Intervention: Acupuncture and related therapies plus CHM |
| 25) Is adding Moxibustion on top of CHM more effective in improving quality of life among adult patients receiving cancer palliative care?  Note: Details of CHM:   1. Wei-chang-an (胃腸安): Pseudostellaria heterophylla (Miq.) Pax [Tai Zi Shen太子參] 12g, Atractylodes macrocephala Koidz. [Bai Zhu白術]12g, Poria [Fu Ling茯苓] 30g, Sargentodoxa cuneata (Oliv.) Rehder & E.H.Wilson [Hong Teng紅藤] 30g, Prunella vulgaris L. [Xia Ku Cao夏枯草] 9g, Smilax china L. [Ba Qia菝葜] 30g, [Lv E Mei綠萼梅] 9g, etc |

Key: CHM, Chinese herbal medicine.

Appendix 3. Demographic characteristics of participants in the face-to-face workshop (n=17)

| Demographics | | Values |
| --- | --- | --- |
| 1. Gender, n (%) | Male | 7 (41.2) |
|  | Female | 10 (58.8) |
| 2. Age, n (%) | 18-29 | 1 (5.9) |
|  | 30-39 | 2 (11.8) |
|  | 40-49 | 5 (29.4) |
|  | 50-59 | 5 (29.4) |
|  | 60 or above | 4 (23.5) |
| 3. Background of  participants, n (%) | Patients with cancer | 5 (29.4) |
|  | Cancer survivors | 6 (35.3) |
|  | Caregivers | 6 (35.3) |
| 4. Duration of receiving Chinese medicine modalities for cancer palliative care* (Range of months) | | 2 months to 102 months |

*Cancer palliative care received by i) patients with cancer, ii) cancer survivors and iii) patients with cancer who were looked after by caregivers in this study.

Appendix 4. List of finalized Chinese medicine clinical research priorities in cancer palliative care identified by international experts versus patients with cancer, cancer survivors and caregivers

| **Final list of research priorities** **generated from**  **a previous international Delphi survey^1^** | **Top ten important research priorities generated from**  **the face-to-face workshop** |
| --- | --- |
| **Study participants:** International experts in cancer palliative care | **Study participants:** Patients with cancer, cancer survivors and caregivers in Hong Kong |
| 1. ***Is adding manual acupuncture on top of opioids more effective in reducing pain among adult patients receiving cancer palliative care?**** | 1. ***Is adding manual acupuncture on top of opioids more effective in reducing pain among adult patients receiving cancer palliative care?**** |
| 1. Is adding manual acupuncture on top of exercise more effective in reducing fatigue among adult patients receiving cancer palliative care? | 1. Is using CHM effective in improving quality of life among adult patients receiving cancer palliative care and chemotherapy? |
| 1. Is adding CHM on top of conventional care (glycerin suppositories/ lactulose syrup) more effective in reducing constipation among adult patients receiving cancer palliative care?^ | 1. Is adding CHM on top of loperamide more effective in reducing stomatitis among adult patients receiving cancer palliative care? |
| 1. Is adding manual acupuncture on top of artificial saliva more effective in reducing xerostomia among p adult patients receiving cancer palliative care? | 1. Is adding CHM on top of conventional care (granulocyte-colony stimulating factor (G-CSF)/ granulocytemacrophage colony stimulating factor (GM-CSF)/ interleukin 3 (IL3)) more effective in improving leukopenia among adult patients receiving cancer palliative care? |
| 1. Is adding manual acupuncture on top of conventional care more effective in reducing anxiety among adult patients receiving cancer palliative care?^ | 1. Is adding manual acupuncture on top of opioids more effective in reducing dyspnea among adult patients receiving cancer palliative care? |
| 1. Is adding electroacupuncture on top of oral vitamin B6 (pyridoxine) supplement more effective in reducing paresthesia and dysesthesia among adult patients receiving cancer palliative care? | 1. Is using CHM on top of progesterone analogs effective in reducing anorexia among adult patients receiving cancer palliative care and chemotherapy? |
| 1. Is adding CHM on top of conventional care (Blood transfusion/ Erythropoiesis-stimulating agents (e.g. Epoetin)) more effective in improving anemia among adult patients receiving cancer palliative care? | 1. Is adding manual acupuncture/ electroacupuncture on top of neurokinin-1 receptor antagonists + serotonin-3 receptor antagonist (5-HT3) and/or dexamethasone more effective in reducing nausea and vomiting, as well as improving quality of life adult patients receiving cancer palliative care? |
| 1. Is acupressure provided by Chinese medicine practitioners more effective than self-acupressure in improving insomnia among adult patients receiving cancer palliative care and chemotherapy? | 1. Is adding CHM on top of conventional care (compression bandaging and manual lymphatic drainage) more effective in reducing limbs edema among adult patients receiving cancer palliative care? |
|  | 1. Is adding CHM on top of conventional care (glycerin suppositories/ lactulose syrup) more effective in reducing constipation among adult patients receiving cancer palliative care?^ |
|  | 1. Is adding manual acupuncture on top of conventional care more effective in reducing anxiety among adult patients receiving cancer palliative care?^ |

**Priority with same ranking across experts and patients*

*^Co-prioritized by experts and patients*

Reference of Appendix 4:

1. Wong CH, Wu IX, Balneaves LG, et al. Prioritizing Chinese Medicine Clinical Research Questions in Cancer Palliative Care: International Delphi Survey. *Journal of Pain and Symptom Management.* 2019;58(6):1002-1014. e1007.
